# A Solitary Fibrofolliculoma in Close Proximity to the Medial Canthus

**DOI:** 10.7759/cureus.48233

**Published:** 2023-11-03

**Authors:** Reshma Sonsale, Wael Hamarneh, Tristan McMullan

**Affiliations:** 1 Ophthalmology, Moorfields Eye Hospital NHS Foundation Trust, London, GBR; 2 Ophthalmology, Northampton General Hospital, Northampton, GBR; 3 Histopathology and Cytopathology, Northampton General Hospital, Northampton, GBR

**Keywords:** histopathology reporting, excision biopsy, solitary lesion, eyelid lesion, fibrofolliculoma

## Abstract

Fibrofolliculomas are benign connective tissue tumours of the hair follicle that typically present as multiple lesions over the head and neck. A solitary fibrofolliculoma is a rare entity and has not been previously described in the canthal region. The authors report an unusual case of a 43-year-old female who was found to have a solitary fibrofolliculoma located in close proximity to the medial canthus following an excision biopsy. Whilst rare, fibrofolliculomas should be considered in the differential diagnosis of an eyelid lesion.

## Introduction

Fibrofolliculomas are benign, perifollicular connective tissue tumours that present as clinically asymptomatic, skin-coloured, dome-shaped, smooth lesions across the head and neck. They were first reported by Birt et al. in 1977 in a case series of 15 people with skin lesions. Histologically, they can be described as abnormal follicles of hair with differentiation of its root sheath and associated infundibular epithelial proliferation [1]. They are typically seen in patients with Birt-Hogg-Dube syndrome, a rare autosomal dominant condition that presents with features such as multiple fibrofolliculomas, pulmonary cysts, spontaneous pneumothoraxes, or renal cancer [2].

A solitary fibrofolliculoma, however, is a rare entity and was first reported in the literature in 1984. In contrast to multiple fibrofolliculomas, a solitary lesion by itself does not have a genetic predisposition and is not associated with other cutaneous abnormalities [3]. There are only four previous cases in the literature of a solitary fibrofolliculoma described on the eyelid [4-7]. Here, we report the first case (to our knowledge) of a solitary fibrofolliculoma located in close proximity to the medial canthus in an otherwise healthy 43-year-old female.

## Case presentation

A 43-year-old female, who worked as a nurse, was referred by her general practitioner (GP) to the oculoplastics service during the COVID-19 pandemic. She had a concern about a lump located below the right lower eyelid and in close proximity to the medial canthus, which had been present for a few months. The referral stated that the lump had been getting larger, and was causing the patient discomfort during the day when they had to wear a mask at work.

By the time this lady was seen in the oculoplastic clinic, the lesion had been present for 12 months. She denied any bleeding, itching, or pain and reported that it was occasionally red. She had no other past ocular history and her past medical history included asthma and hypothyroidism.

**Figure 1:**
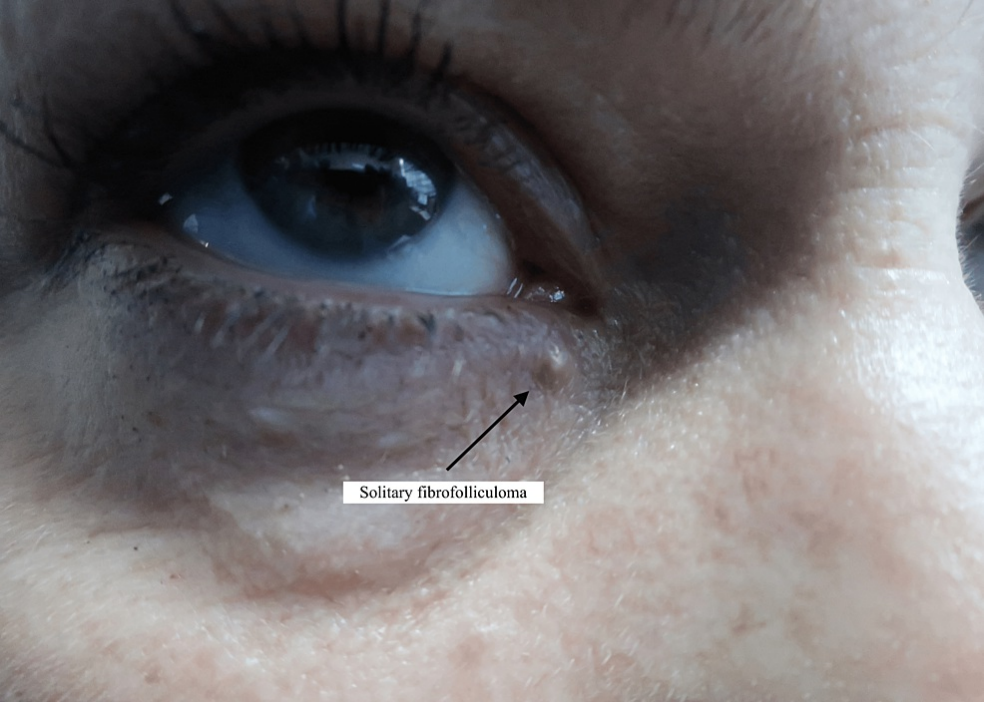
A solitary fibrofolliculoma in the medial canthus

On examination, the lesion was described as a 3 mm cystic lesion (Figure 1).

**Figure 2:**
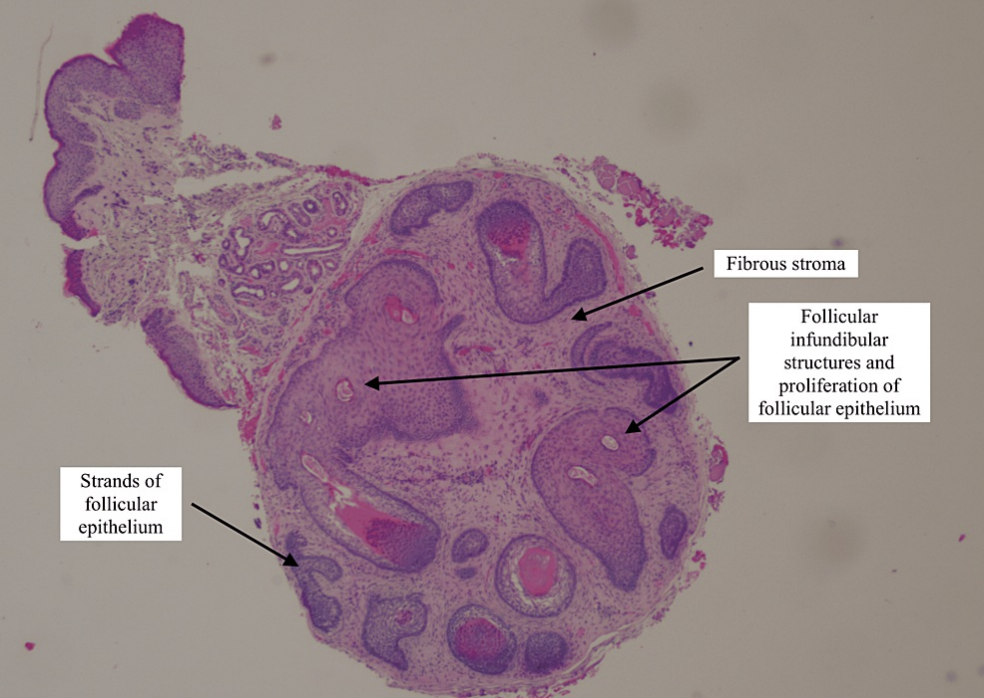
A low-power (10x magnification) histology slide image of the solitary fibrofolliculoma

**Figure 3:**
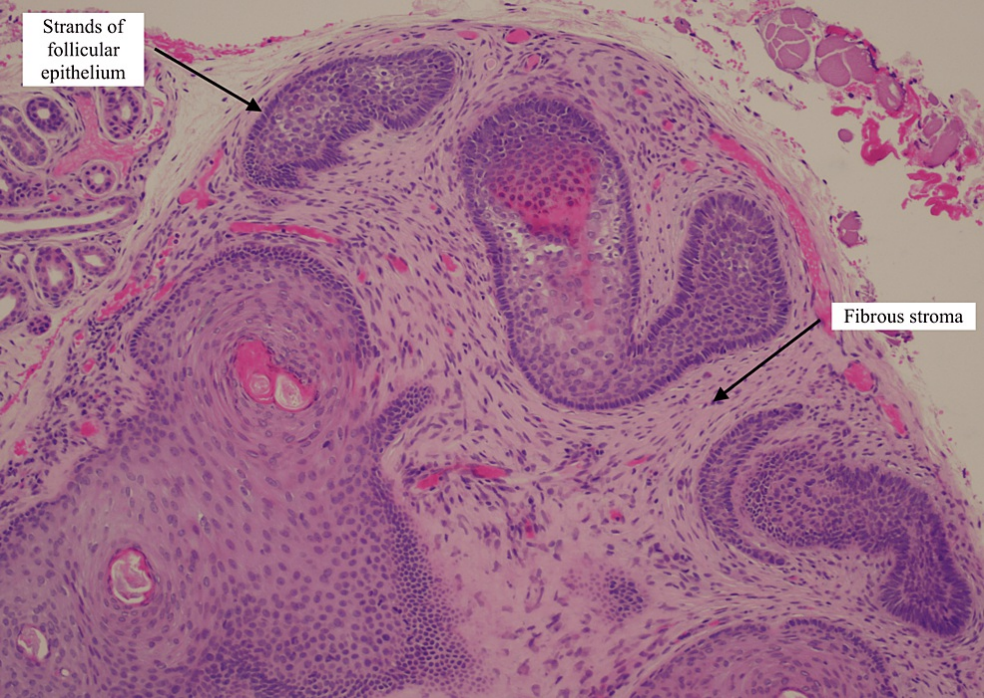
A low-power (20x magnification) histology slide image of the solitary fibrofolliculoma

The rest of her ocular examination was unremarkable. Based on these findings, a possible diagnosis of a cyst of Zeiss was suggested. Since the location of the lesion was susceptible to trauma, she was listed for an excisional biopsy and subsequently underwent a right medial canthal lesion biopsy under local anaesthesia. The histology referral specifically queried whether the specimen was a naevus or a basal cell carcinoma (BCC), which represents possible differential diagnoses. The final histology result reported a 2 mm skin nodule with skin sections of underlying fibrofolliculoma with no dysplasia, melanocytic proliferation, or malignancy seen. The histology slides from this patient show skin with underlying circumscribed unencapsulated lesion composed of epidermal strands set in a fibrotic stroma, also known as a hamartoma of the hair follicle (Figures 2, 3).

The patient was followed up in an outpatient clinic where the benign nature of the diagnosis was explained to her.

## Discussion

Cases of a solitary fibrofolliculoma on the eyelid are rare; since first being reported in 2007, there have been a total of four cases that have been published in MEDLINE (Table 1).

**Table 1:** Cases of eyelid solitary fibrofolliculomas in the literature

	Publication year	Age	Gender	Location	Symptom duration	Clinical diagnosis	Treatment
Chang et al. (2007) [4]	2007	37	F	Upper eyelid	12 months	Chalazion	Excision biopsy
Diez-Montero et al. (2020) [5]	2020	72	M	Lower eyelid	Not specified	Lipoma	Excision biopsy
Wang et al. (2020) [6]	2020	68	F	Upper eyelid	5 years	N/A	Shave excision
Mishra et al. (2021) [7]	2021	50	M	Lower eyelid	6 months	Sebaceous gland carcinoma	Excision biopsy

We reviewed the previously published cases and found that the age of patients ranged from 37 years to 72 years with no clear gender preference. There was no clear trend towards either eyelid, with two of these cases reported on the upper eyelid and two on the lower eyelid. Our report describes the first case of a solitary fibrofolliculoma found in the canthal region. Amongst the published case reports, symptom duration varied from a few months to several years. The commonest diagnostic and treatment modality, including the preferred option in the present case, was excision biopsy.

The majority of eyelid tumours are of cutaneous origin, which includes benign epithelial lesions, BCCs and melanocytic tumours [8]. Fibrofolliculomas are classified as hamartomas and are made up of perifollicular connective tissue and a hair follicular epithelial component [9]. Clinically, they appear as small dome-shaped papules, yet in our case, the examination findings of a cystic lesion with no sinister features led to an initial diagnosis of a cyst of Zeiss. This was a more likely diagnosis given the eyelids have a rich supply of these sebaceous-filled glands [8]. Some sinister eyelid lesion criteria include ulceration, infiltration, and loss of eyelashes, which help to indicate which lesions may need biopsy and/or removal [10]. Despite our patient not exhibiting any of the aforementioned features, it was felt an excision biopsy was the most appropriate treatment method since it was in a location that was susceptible to trauma. However, if the lesion had not presented in this location, an excision biopsy would likely not have been requested given the lesion’s otherwise benign features.

Since solitary fibrofolliculomas are rare and need histological assessment to confirm diagnosis, they can be easily misdiagnosed without biopsy as could have been in this case. Chang et al. described this condition in the case of a 37-year-old female patient, noting that the lesion was present for five years and was initially misdiagnosed as a chalazion [4]. This kept recurring despite receiving incision and curettage as treatment, leading the team to perform an excision biopsy confirming the diagnosis of a fibrofolliculoma. In a more recent case reported by Mishra et al. [7], a 50-year-old male patient presented with a slowly enlarging lesion on the lower eyelid for six months and a clinical diagnosis of a sebaceous gland carcinoma was suspected, which led to an excision biopsy and consequent histopathological examination.

## Conclusions

To conclude, we present a case of a lesion arising in close proximity to the medial canthus showing the histological features of a fibrofolliculoma. Whilst rare, a diagnosis of solitary fibrofolliculoma may be included in the differential diagnosis when a similar lesion is observed. An excision biopsy with histological assessment is an effective way to confirm the diagnosis, as well as exclude possible malignant causes.
